# Long non-coding RNA H19 in atherosclerosis: what role?

**DOI:** 10.1186/s10020-020-00196-w

**Published:** 2020-07-22

**Authors:** Xian Shi, Ya-Ting Wei, Heng Li, Ting Jiang, Xi-Long Zheng, Kai Yin, Guo-Jun Zhao

**Affiliations:** 1 School of Medicine, Guilin Medical University, Guilin, 541100 Guangxi China; 2 Institute of Cardiovascular Research, Key Laboratory for Arteriosclerology of Hunan Province, University of South China, Hengyang, 421001 Hunan China; 3 The Sixth Affiliated Hospital of Guangzhou Medical University, Qingyuan City People’s Hospital, Qingyuan, 511518 Guangdong China; 4 Department of Biochemistry and Molecular Biology, The Libin Cardiovascular Institute of Alberta, The University of Calgary, Health Sciences Center, Calgary, AB Canada; 5 Key Laboratory of Molecular Targets and Clinical Pharmacology, School of Pharmaceutical Sciences, Guangzhou Medical University, Guangzhou, 511436 Guangdong China; 6 Center for Diabetic Systems Medicine, Guangxi Key Laboratory of Excellence, The Second Affiliated Hospital of Guilin Medical University, Guilin, 541100 Guangxi China

**Keywords:** LncRNA-H19, Angiogenesis, Lipid metabolism, Inflammatory response, Atherosclerosis

## Abstract

Atherosclerosis (AS) is widely accepted to be a multistep pathophysiological process associated with several other processes such as angiogenesis and inflammatory response. Long non-coding RNAs (lncRNAs) are non-protein coding RNAs (more than 200 nucleotides in length) and can regulate gene expression at the transcriptional and post-transcriptional levels. Recent studies suggest that lncRNA-H19 plays important roles in the regulation of angiogenesis, adipocyte differentiation, lipid metabolism, inflammatory response, cellular proliferation and apoptosis. In this review, we primarily discuss the roles of lncRNA-H19 in atherosclerosis-related pathophysiological processes and the potential mechanisms by which lncRNA-H19 regulates the development of atherosclerosis, to help provide a better understanding of the biological functions of lncRNA-H19 in atherosclerosis.

## Introduction

Atherosclerosis (AS) drives cardiovascular disease, which is one of the main causes of mortality in the world (Libby et al. 2019; Barquera et al. 2015). Atherogenesis is a slowly progressive process that is characterized by multifocal structural alterations in the wall of large and medium-sized arteries and the subsequent formation of atherosclerotic plaques. Many pathophysiological processes are mechanistically involved in the pathogenesis and development of atherosclerosis, such as angiogenesis, inflammatory and immune responses, adipogenesis, lipid metabolism, cellular proliferation and apoptosis. These processes are critical for the development of atherosclerosis and ultimately trigger thrombotic plaque complications, such as myocardial infarction (MI), stroke, and cardiovascular death (Libby et al. 2019; Camaré et al. 2017; Ross 1999).

Long non-coding RNAs (lncRNAs) are widely classified as transcripts > 200 nucleotides with limited coding potential (Klattenhoff et al. 2013). Numerous functions of lncRNAs in many biological activities have been found, such as: (i) serving as precursors for shorter functional RNAs as exemplified by primary transcripts for microRNAs (miRNAs); (ii) regulating transcription, translation, imprinting, genome rearrangement and chromatin modification; (iii) regulating protein activities; (iv) producing scaffolds for higher-order complexes, such as Polycomb repressive complex 2 (PRC2); (v) working as competing endogenous RNAs (ceRNAs) or natural miRNA sponges. All RNA transcripts that contain miRNA-binding sites can communicate with and regulate each other by competing specifically for shared miRNAs and thus lncRNAs can impact target gene mRNA expression by acting as miRNA molecular sponges (Fatica and Bozzoni 2014; St Laurent et al. 2015; Tay et al. 2014).

Recently, emerging evidence shows that lncRNAs are crucial regulators in many pathophysiological processes of atherosclerosis (Aryal and Suarez 2019; Kim and Kook 2019; Yu and Wang 2018). For example, lncRNA-p21 expression, which is significantly decreased in atherosclerotic plaques of ApoE^−/−^ mice, suppresses proliferation and facilitates apoptosis of vascular smooth muscle cells (VSMCs) by increasing p53 activity (Wu et al. 2014). In addition, the lncRNA SMILR, as another example, regulates the proliferation of VSMCs, and its expression is significantly upregulated in unstable human atherosclerotic plaques (Ballantyne et al. 2016).

The *H19* gene, which encodes lncRNA-H19, is localized near the telomeric region of chromosome 11p15, within a unique locus shared with the insulin-like growth factor 2 *(IGF2)* gene. The *H19* gene is 2.3 kb in length and is highly evolutionarily conserved, suggesting that it may have some important biological functions (Hurst and Smith 1999). Based on their functions, lncRNAs can be classified as ncRNA-activating, ceRNAs and precursors for shorter functional RNAs as exemplified by primary transcripts for miRNAs and piRNAs (St Laurent et al. 2015). Importantly, lncRNA-H19 can act as a miRNA primary transcript to generate miRNA or as a ceRNA to sponge miRNA (St Laurent et al. 2015; Cai and Cullen 2007; Yan et al. 2015). Some previous trials found that lncRNA-H19 is highly expressed in human atherosclerotic plaques and injured carotid arteries in rat model but was barely expressed in normal coronary arteries (Han et al. 1996; Kim et al. 1994). Recent studies have shown that increased plasma level of lncRNA-H19 is associated with an increased risk of coronary artery disease (Bitarafan et al. 2019; Zhang et al. 2017). In patients with atherosclerosis, a high level of lncRNA-H19 is detected and overexpression of lncRNA-H19 promotes proliferation and inhibits apoptosis of VSMCs (Pan 2017). Furthermore, Huang et al. reported that overexpression of lncRNA-H19 contributes to the occurrence of atherosclerosis (Huang et al. 2019). These findings indicate that lncRNA-H19 may be involved in the onset and progression of atherosclerosis.

In this review, we summarized the current studies and discussed the roles of lncRNA-H19 in atherosclerosis to demonstrate the potential value of lncRNA-H19 in atherosclerosis therapy and provide a basis for further investigations.

## LncRNA-H19 and the pathophysiology of atherosclerosis

The development of native atherosclerosis is mechanistically considered to be the consequence of dysregulation of numerous biological phenomena including angiogenesis, adipogenesis, lipid metabolism, inflammatory response, cellular proliferation and apoptosis (Camaré et al. 2017; Ross 1999). Many studies have shown that lncRNA-H19 is involved in these phenomena via various mechanisms.

### LncRNA-H19 regulates angiogenesis

Angiogenesis significantly influences plaque growth and instability in atherosclerotic lesions. Increased neovascularization enhances the local supply of nutrients and O_2_ in atherosclerotic areas, thereby promoting plaque progression and remodeling. The incomplete maturation and the fragility of neocapillaries likely promote intraplaque hemorrhages, leading to plaque instability and rupture that are often associated with athero-thrombotic events (Camaré et al. 2017; Falk 2006).

Some studies have shown that lncRNA-H19 participates in vascular physiopathology and angiogenesis. It was reported that inhibition of lncRNA-H19 results in dramatic reductions in endothelial cell (EC) growth and capillary-like structure formation (Voellenkle et al. 2016). Moreover, a recent study demonstrated that endothelial-specific knockdown of lncRNA-H19 impairs angiogenesis while exogenous lncRNA-H19 partially rescues this effect (Hofmann 2018).

Vascular endothelial growth factor (VEGF) is considered as one of the crucial factors associated with angiogenesis (Di Stefano et al. 2009). The VEGF family includes several members: VEGF-A, −B, −C, −D, −E, and -F and placental growth factor (PlGF) (Hoeben et al. 2004). In aortic tissues of atherosclerotic mice, knockdown of lncRNA-H19 prevents intraplaque angiogenesis and downregulates the expression of the angiogenesis-related factors matrix metalloproteinase 2 (MMP-2) and VEGF (Yang et al. 2019). In contrast, lncRNA-H19 overexpression recruits CCCTC-binding factor (CTCF) to repress the polycystic kidney disease 1 *(PKD1)* gene (Yang et al. 2019), mutations of which may lead to angiogenesis (Li et al. 2003). VEGF-A has been certified as a target of microRNA-199a-5p (miR-199a-5p) (Hsu et al. 2014). Interestingly, lncRNA-H19 serves as a ceRNA to inhibit miR-199a-5p, resulting in the upregulation of VEGF-A expression. Moreover, lncRNA-H19 enhances the survival of mesenchymal stem cells and their angiogenic potential in vitro ^31^. Some studies have shown that VEGF-A is also a target of miR-29a and miR-29c (Chen et al. 2014; Liu et al. 2017). Knockdown of lncRNA-H19 suppresses glioma-induced EC proliferation, migration and tube formation by upregulating miR-29a (Jia et al. 2016). In addition, lncRNA-H19 can enhance corneal neovascularization by binding directly to miR-29c, which negatively regulates VEGF-A (Sun et al. 2019a). Moreover, Zhu et al. revealed that lncRNA-H19 overexpression exerts pro-angiogenic effects in human dermal vascular endothelial cells (HMEC-1) (Zhu et al. 2019a). LncRNA-H19 overexpression also increases the protein levels of VEGF and endothelial NO synthase (eNOS) in HMEC-1 cells, suggesting that lncRNA-H19 promotes tube formation by regulating VEGF and eNOS. Further experiments showed that the underlying mechanism is associated with lncRNA-H19-mediated downregulation of miR-181a, and subsequent activation of the c-Jun N-terminal kinase (JNK) and AMP-activated protein kinase (AMPK) pathways (Zhu et al. 2019a). Human amniotic mesenchymal stem cells (HAMSCs) express and secrete significantly high levels of representative pro-angiogenic factors including VEGF-A (Kim et al. 2012). A recent study found that lncRNA-H19 knockdown in HAMSCs suppresses angiogenesis. Mechanistically, lncRNA-H19 interacts with the histone methyltransferase enhancer of zeste homolog 2 (EZH2). LncRNA-H19 knockdown inhibits EZH2 from recruiting methyl groups to the promoter region of the angiogenesis inhibitor vasohibin1 *(VASH1)* gene, thus increasing VASH1 expression and secretion in HAMSCs, and suppressing angiogenesis (Yuan et al. 2019). In addition, Hofmann et al. showed that lncRNA-H19 is decreased during aging and controls EC senescence, proliferation and angiogenic sprouting by inhibiting the activation of signal transducer and activator of transcription 3 (STAT3) (Hofmann et al. 2019), while STAT3 activation upregulates the expression of VEGF (Kujawski et al. 2008; Niu et al. 2002).

In summary, these studies suggest that lncRNA-H19 promotes angiogenesis and accelerates the development of atherosclerosis.

### LncRNA-H19 regulates adipocyte differentiation

White adipose tissue (WAT), brown adipose tissue (BAT) and perivascular adipose tissue (PVAT) play differential roles in atherosclerosis. WAT, acting as a lipid sink, prevents the accumulation of lipids in circulation but active BAT helps combust lipids. PVAT has properties of both WAT and BAT. Evidence suggests a critical role for PVAT in regulating the focal inflammatory state and vessel homeostasis via pro-atherogenic or anti-atherogenic mechanisms such as releasing inflammatory cytokines and adipokines, depending on the PVAT state in health and disease (Ahmadieh et al. 2020; van Dam et al. 2017; Verhagen and Visseren 2011).

Bone marrow mesenchymal stem cells (BMSCs) are precursor cells of adipocytes (Pittenger et al. 1999), and lncRNA-H19 is a primary miRNA precursor for miR-675 (Smits et al. 2008). Huang et al. found that in human BMSCs, lncRNA-H19 and lncRNA-H19-derived miR-675 inhibits BMSCs differentiation into adipocytes. Mechanistically, miR-675 directly binds to the 3′ UTRs of class II histone deacetylase (HDAC) 4–6 transcripts and downregulates their expression (Huang et al. 2016). Class II HDACs play an essential role in adipocyte differentiation (Nebbioso et al. 2010), thus their downregulation inhibits adipocyte differentiation of human BMSCs (Huang et al. 2016). In a more recent trial using mouse BMSCs, knockdown of lncRNA-H19 markedly increases miR-188 expression. MiR-188 overexpression promotes adipocyte differentiation of mouse BMSCs by directly removing the effects of ligand-dependent corepressor (LCoR) (Wang et al. 2018), and LCoR is a negative regulator of adipogenesis (Cao et al. 2017). Therefore, through this lncRNA-H19/miR-188/LCoR pathway, lncRNA-H19 knockdown subsequently induces adipocyte differentiation in mouse BMSCs (Wang et al. 2018). Taken together, these findings suggest that lncRNA-H19 may have a negative correlation with adipocyte differentiation of BMSCs by regulating its targeted genes.

### LncRNA-H19 regulates lipid metabolism

The lipid accumulation occupies an important position in the progression of atherosclerosis. For example, foam cell formation is a vital process in atherogenesis and involves phagocytosis of matrix-retained lipoproteins and fluid-phase pinocytosis of aggregated lipoproteins by macrophages (Libby et al. 2019; Weber and Noels 2011). Han et al. observed that lncRNA-H19 knockdown in foam cells counteracts the increased amount of triglyceride (TG), total cholesterol (TC), and low density lipoprotein-cholesterol (LDL-C) and the decreased amount of high density lipoprotein-cholesterol (HDL-C). Moreover, the Oil red O staining revealed that lncRNA-H19 knockdown decreases lipid accumulation. These results suggested that lncRNA-H19 induces lipid metabolic disorders in foam cells by suppressing lipid metabolism and increasing lipid accumulation, which contribute to the progression of atherosclerosis. In addition, it was found that miR-130b is downregulated in foam cells compared with the normal macrophages (Han et al. 2018), and is also involved in lipid metabolic disorders (Lv et al. 2015). Therefore, miR-130b may be a potential target of lncRNA-H19, which is involved in lipid metabolism and atherosclerosis (Han et al. 2018).

Nonalcoholic fatty liver disease (NAFLD) is strongly associated with atherosclerosis (Sookoian and Pirola 2008; Zheng et al. 2018). Peroxisome proliferator-activated receptor γ (PPARγ) was reported to be correlated with NAFLD (Zhu et al. 2019b). In hepatocytes, it was reported that miR-130a directly binds with lncRNA-H19 and PPARγ. By directly upregulating miR-130a, lncRNA-H19 knockdown inhibits PPARγ expression to alleviate lipid accumulation in hepatocytes. Hence, lncRNA-H19 promotes hepatic lipogenesis via the lncRNA-H19/miR-130a/PPARγ axis (Liu et al. 2019). Sterol regulatory element-binding protein 1c (SREBP1c) is an endoplasmic reticulum membrane-bound protein that functions as a transcription factor in the liver and the induction of lipogenesis is mainly controlled by SREBP1c (Watanabe et al. 2004). Liu et al. found that overexpression of lncRNA-H19 in hepatocytes increases the endogenous nuclear SREBP1 protein, thereby enhancing lipid accumulation. Further analysis showed that lncRNA-H19 interacts with polypyrimidine tract-binding protein 1 (PTBP1, also known as PTB, or hnRNP I) to enhance the binding of PTBP1 to SREBP1c mRNA. The combination of lncRNA-H19 and PTBP1 increases the stability and transcriptional activity of SREBP1c mRNA (Liu et al. 2018).

BAT activation lowers plasma TG and cholesterol levels, attenuating atherosclerosis (Berbee et al. 2015). Schmidt et al. reported that lncRNA-H19 possesses positive effects on the differentiation and mature fat cell function in BAT (Schmidt et al. 2018). This group showed that overexpression of lncRNA-H19 enhances, while its silence impairs adipogenesis, oxidative metabolism and mitochondrial respiration in brown adipocytes but not white adipocytes. Moreover, they found that lncRNA-H19 recruits the chromatin modifier methyl-CpG–binding domain protein 1 (MBD1) to form H19-MBD1 chromatin modifier complexes. These complexes specifically repress paternally expressed genes (PEGs) in brown adipocytes (Schmidt et al. 2018), and PEGs negatively affect BAT (Peters 2014), thereby lncRNA-H19 acting as the selective PEG gatekeeper in BAT (Schmidt et al. 2018). This finding may also shed light on the role of lncRNA-H19 in adipogenesis.

Collectively, lncRNA-H19 appears to play an important role in regulating lipid metabolism. In this way, lncRNA-H19 participates in atherosclerosis and its function is possibly tissue-specific while the underlying mechanisms need to be further studied.

### LncRNA-H19 regulates inflammatory response

The inflammatory response is critically involved in the pathogenesis of atherosclerosis (Libby et al. 2019; Ross 1999; Weber and Noels 2011). Several mechanisms underlying the roles of inflammation in atherosclerosis have been identified, such as inflammatory activation of ECs, foam cell formation, foam cell secretion of inflammatory cytokines, and macrophage cell death (Geovanini and Libby 2018).

Previous studies have revealed that lncRNA-H19 is involved in several kinds of inflammatory responses. For example, in a rat model of diabetic cardiomyopathy, overexpression of lncRNA-H19 reduces the concentrations of inflammatory cytokines in myocardial tissues (Li et al. 2016). Moreover, Hu et al. demonstrated that pro-inflammatory factors (IL-1β, IL-6 and TNF-α) are reduced after the knockdown of lncRNA-H19 in lipopolysaccharide (LPS)-treated C28/I2 cells (Hu et al. 2019). Hence, there is a good reason to propose that lncRNA-H19 plays a role in inflammatory responses.

As a major transcription factor of inflammatory responses, nuclear factor-κB (NF-κB) was first discovered in 1986 (Sen and Baltimore 1986). Many NF-κB activators and NF-κB-regulated genes have been identified to be involved directly or indirectly in the process of atherosclerosis (Pamukcu et al. 2011).

Compelling evidence has revealed that mitogen-activated protein kinase (MAPK) signaling is a vital regulator of NF-κB-mediated inflammatory responses (Yuan et al. 2014; Shi et al. 2016; He et al. 2018). Overexpression of lncRNA-H19 leads to an increase of p38 and p65 in human umbilical vein endothelial cells (HUVECs), which are both key factors in the MAPK and NF-κB signaling pathways (Pan 2017). Similar results were reported in a recent trial also using HUVECs, after overexpression of lncRNA-H19, the NF-κB pathway is activated, p38 and p65 are increased (Li et al. 2019a), which further supports that lncRNA-H19 can increase the NF-κB-mediated inflammatory responses in vascular ECs.

In addition, knockdown of lncRNA-H19 in foam cells effectively decreases the expression of pro-inflammatory factors (TNF-α and IL-β) and increases the expression of anti-inflammatory factors (IL-4 and IL-10) (Han et al. 2018). The authors hypothesized that miR-130b is a target of lncRNA-H19. By upregulating miR-130b expression, lncRNA-H19 knockdown removes the facilitating effects of the miR-130b inhibitor on inflammatory responses, thereby lncRNA-H19 knockdown alleviates the inflammatory responses (Han et al. 2018). The miR-130 family negatively regulates metabolism-related inflammatory processes through several pathways including the NF-κB signaling pathway (Song et al. 2016; Zheng et al. 2016). Thus, it can be inferred that lncRNA-H19 plays a pro-inflammatory role via the potential lncRNA-H19/miR-130b/NF-κB pathway.

NF-κB possesses a causative function in inflammation, when in response to inflammatory signals, it promotes interleukin-6 (IL-6) expression by downregulating microRNA let-7. IL-6, which is a pro-inflammatory cytokine, activates NF-κB, thereby completing a positive feedback loop of the NF-κB/let-7/IL-6 pathway (Iliopoulos et al. 2009). Interestingly, in addition to regulating NF-κB, lncRNA-H19 can directly target let-7 to regulate inflammatory responses. In a recent study using a mouse model of abdominal aortic aneurysm (AAA), by sponging let-7a as a ceRNA, overexpression of lncRNA-H19 in VSMCs increases IL-6 expression, and ultimately promotes vascular inflammation and induces AAA formation. Moreover, the authors detected high levels of lncRNA-H19 in human and mouse AAA tissue samples (Sun et al. 2019b). Furthermore, Cao et al. showed that lncRNA-H19/let-7 axis participates in the regulation of oxygenized low density lipoprotein (ox-LDL)-induced EC injury. LncRNA-H19 knockdown in HUVECs reduces ox-LDL-induced secretion of inflammatory cytokines, such as IL-6 and TNF-α. The underlying mechanism may be that lncRNA-H19 inhibits periostin expression at least partially by sponging let-7 (Cao et al. 2019), and periostin acts as a regulator of inflammatory diseases including atherosclerosis (Koh et al. 2016; Schwanekamp et al. 2016). Taken together, these studies demonstrate a possible lncRNA-H19/let-7/IL-6 pathway for lncRNA-H19 regulation of atherosclerosis-related inflammatory responses, suggesting that lncRNA-H19 regulates inflammatory responses through several mechanisms.

### LncRNA-H19 regulates cellular proliferation and apoptosis

The major cells involved in atherosclerosis such as VSMCs and ECs, are considered to undergo abnormal cellular proliferation and apoptosis to develop atherosclerosis. Aberrant apoptosis of VSMCs, for example, promotes both atherogenesis and multiple features of plaque instability (Ross 1999; Bennett et al. 2016).

Overexpression of lncRNA-H19 in VSMCs and HUVECs induces an increase in proliferation and a decrease in apoptosis (Pan 2017). Moreover, it has been reported that ox-LDL upregulates the expression of lncRNA-H19 in VSMCs and promotes the proliferation of VSMCs (Xu et al. 2015). Thus it can be inferred that lncRNA-H19 may have a role in cellular proliferation and apoptosis. In a recent trial using VSMCs and HUVECs, lentivirus-mediated lncRNA-H19-forced expression upregulates acid phosphatase 5 (ACP5) protein levels and subsequently promotes cellular proliferation and suppresses apoptosis. The authors hypothesized that ACP5, as a direct target of lncRNA-H19, causes atherosclerosis by affecting cellular proliferation and apoptosis (Huang et al. 2019).

Many studies have reported that lncRNA-H19 functions as a ceRNA to sponge its target miRNAs such as microRNA let-7 (Hsu et al. 2014; Sun et al. 2019b; Cao et al. 2019). Sun et al. found that lncRNA-H19 sponges let-7a, which binds to the 3′ UTR of cyclin D1 mRNA to exert negative regulation; therefore, lncRNA-H19 positively regulates cyclin D1, which is a key factor promoting the proliferation of VSMCs. Hence, lncRNA-H19 promotes the proliferation of VSMCs through the lncRNA-H19/let-7a/cyclin D1 axis (Sun et al. 2019c). Similarly, it was reported that lncRNA-H19 sponges let-7b to stimulate the expression of Ang II type 1 receptor (AT_1_R), which is a critical regulator of vasoconstriction and proliferation of arteries. Through the lncRNA-H19/let-7b/AT_1_R axis, lncRNA-H19 also promotes the proliferation of VSMCs (Su et al. 2018). In addition, Zhang et al. found that lncRNA-H19 functions as a ceRNA of miR-148b to enhance WNT1 expression, and the WNT/β-catenin signaling pathway is a vital pathway in the proliferation and apoptosis of VSMCs. Thus, lncRNA-H19 could facilitate proliferation and inhibit apoptosis of VSMCs through the lncRNA-H19/miR-148b/WNT/β-catenin axis (Zhang et al. 2018a). Taken together, these trials indicate that lncRNA-H19 promotes the proliferation and suppresses apoptosis of VSMCs by acting as a ceRNA to regulate its target genes.

In addition to working as a ceRNA, lncRNA-H19 can also regulate the proliferation and apoptosis of VSMCs by generating miR-675. Lv et al. found that lncRNA-H19 was overexpressed in balloon-injured carotid arteries. Their further results suggested that overexpression of lncRNA-H19 accelerates human aortic VSMC (HA-VSMC) proliferation in a miR-675-dependent manner. They identified that phosphatase and tensin homology deleted on chromosome ten (PTEN) is a target of miR-675 and PTEN is a well-known tumor suppressor that mediates VSMC proliferation. Hence, a lncRNA-H19/miR-675/PTEN axis was uncovered, through which lncRNA-H19 promotes the proliferation of VSMCs (Lv et al. 2018). Also via a miR-675-dependent pathway, another study reported that in balloon-injured rat carotid arteries, loss of lncRNA-H19 led to an increase of VSMC apoptosis. The authors found that H19-derived miR-675-5p targets and downregulates Mitofusin-2 (Mfn2) (Xu and Sun 2018), which has pro-apoptotic and anti-proliferative effects (Jin et al. 2011; Wang et al. 2012). However, in a study using a rat model of AAA, the authors found miR-675-independent pro-apoptosis effects of lncRNA-H19 on HA-VSMCs. Mechanistically, lncRNA-H19 enhances hypoxia-inducible factor 1α (HIF1α) expression and retains it within the cytoplasm. Increased cytoplasmic HIF1α directly interacts with mouse double minute 2 homolog (Mdm2) and inhibits Mdm2-mediated reduction of p53, leading to downregulation of the antiapoptotic mediator B cell lymphoma 2 (Bcl-2) and upregulation of the proapoptotic protein Bcl-2 associated X (BAX) (Li et al. 2018). Therefore, it is possible that lncRNA-H19 induces pro-proliferation and anti-apoptosis effects on VSMCs by generating miR-675 but it also induces pro-apoptosis effects on VSMCs when it functions via other mechanisms independent of miR-675.

Additionally, homocysteine (Hcy) induces proliferation of VSMCs. It has been reported that Hcy treatment results in the demethylation of differentially methylated regions between the *H19* gene and *IGF2* gene, which increases the expression of *H19* and decreases the expression of *IGF2*. The proliferation of VSMCs induced by Hcy may be related to this mechanism (Li et al. 2009).

## LncRNA-H19 and atherosclerosis-related cardiac dysfunction

Atherosclerosis is the major cause of coronary artery disease, leading to myocardial ischemia and infarction (Barquera et al. 2015; de Valk and Marx 1999). In addition to the regulation of vascular function, lncRNA-H19 has been suggested to participate in cardiomyocyte injury induced by ischemia/reperfusion (I/R) or infarction (Zhang et al. 2020; Li et al. 2019b).

The mRNA level of lncRNA-H19, which is significantly increased in acute myocardial infarction (AMI) patients, is positively correlated with cardiac biomarkers, such as creatine kinase (CK), suggesting a potential role for lncRNA-H19 in AMI diagnosis (Wang et al. 2019). It was found that lncRNA-H19 expression is significantly decreased in the infarcted myocardium of mice, and overexpression of lncRNA-H19 in AMI mice reduces infarct size and improve cardiac function by activating autophagy (Zhou et al. 2018). Using a rat model of AMI, Zhang et al. revealed that lncRNA-H19 sponges miR-22-3p to ameliorate AMI-induced myocardial damage by upregulating lysine (K)-specific demethylase 3A (KDM3A) (Zhang et al. 2020). It was also reported that lncRNA-H19 protects H9C2 cells against hypoxia-induced injury by sponging miR-139, which targets Sex determining region Y (SRY)-related high-mobility group box 8 (Sox8) (Gong et al. 2017). In addition, Choong et al. demonstrated that lncRNA-H19 and its interacting protein Y-box-binding protein-1 (YB-1) are involved in extracellular matrix (ECM) regulation during cardiac remodeling after infarction, and lncRNA-H19 directly antagonizes YB-1 under hypoxia, resulting in de-repression of Collagen 1A1 expression and cardiac fibrosis (Choong et al. 2019).

With regard to I/R injury, it was reported that rat lncRNA-H19 levels were increased following myocardial I/R injury (Rajagopalan et al. 2017). Li et al. showed that lncRNA-H19 upregulates Bcl-2 expression by sponging miR-877-3p and consequently alleviate myocardial I/R in mice and cardiomyocyte injury induced by H_2_O_2_ (often utilized to establish an in vitro model for I/R injury) (Li et al. 2019b). Similarly, lncRNA-H19 sponges miR-103 and miR-107, suppressing their expression and targeting Fas-associated protein with death domain (FADD) to antagonize cardiomyocyte necrosis in H_2_O_2_-induced H9C2 cells and a mouse I/R model (Wang et al. 2015). Furthermore, Chen et al. found that lncRNA-H19 protects against H_2_O_2_-induced cardiomyocyte injury by increasing the stability of nucleolin protein (Chen et al. 2020). In another study using hypoxic postconditioning (H/Post)-treated aged cardiomyocytes, lncRNA-H19 inhibits aged myocardial apoptosis and relieve H/Post-associated injury by sponging miR-29b-3p targeting cellular inhibitor of apoptosis protein 1 (cIAP1) (Zhang et al. 2019). However, a recent study showed that knockdown of lncRNA-H19 markedly improves the alterations of cardiac structure and function in myocardial I/R, at least partially due to the regulation of the miR-675/PPARα axis (Luo et al. 2019).

## Conclusion

**Fig. 1:**
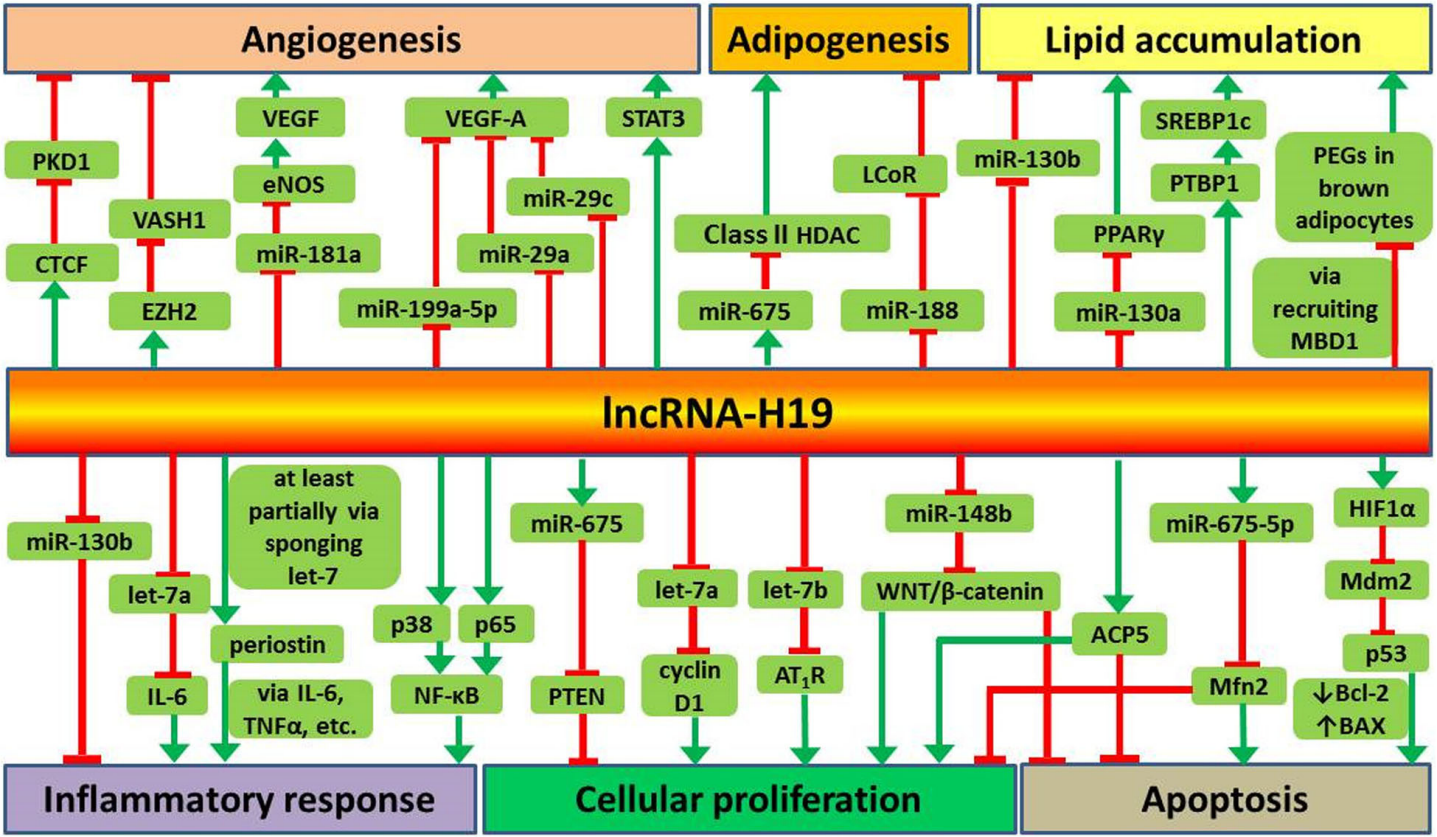
The role of lncRNA-H19 and its targets in atherosclerosis-related metabolisms. LncRNA-H19 may regulate several targets implicated in the dysregulation of angiogenesis, adipogenesis, lipid accumulation, inflammatory responses, cellular proliferation and apoptosis.: promote,: inhibit

LncRNA-H19 plays important roles in the onset, development and progression of atherosclerosis by regulating its targets. Both clinical and laboratory research data suggest emerging roles for lncRNA-H19 and its pathways in various functions (Table 1), which are involved in the regulation of angiogenesis, adipocyte differentiation, lipid metabolism, inflammatory responses, cellular proliferation and apoptosis (Fig. 1).

**Table 1:** The pathways of lncRNA-H19 in atherosclerosis-related pathophysiological processes

Pathway	Function	Experiment	Experimental Material	Ref.
lncRNA-H19/CTCF/PKD1	pro-angiogenesis	in vivo	aortic tissues from ApoE^−/−^ knockout mice	(Yang et al. 2019)
lncRNA-H19/miR-199a-5p/VEGF-A,	pro-angiogenesis	in vitro	BMSCs from mice	(Hou et al. 2018)
lncRNA-H19/miR-29a/VEGF-A,	pro-angiogenesis	in vitro	glioma tissue specimens from patients diagnosed with glioma undergoing surgical resection, human brain microvascular endothelial cell line	(Jia et al. 2016)
lncRNA-H19/miR-29c/VEGF-A	pro-angiogenesis	in vitro and in vivo	vascularized corneas from patients, rat model of CNV, HUVECs	(Sun et al. 2019a)
lncRNA-H19/miR-181a/eNOS/VEGF	pro-angiogenesis	in vitro	HMEC-1 cells	(Zhu et al. 2019a)
lncRNA-H19/EZH2/VASH1	pro-angiogenesis	in vitro and in vivo	subcutaneous model in nude mice, HAMSCs, HUVECs	(Yuan et al. 2019)
lncRNA-H19/STAT3	pro-angiogenesis	in vitro*,* in vivo and ex vivo	carotid plaques from patients, endothelial-specific inducible lncRNA-H19 deficient mice and control littermates, aortic rings from young and aged mice, monocytes, HUVECs, hCoAECs	(Hofmann et al. 2019)
lncRNA-H19/miR-675/Class II HDAC	anti-adipogenic	in vitro	BMSCs from human	(Huang et al. 2016)
lncRNA-H19/miR-188/LCoR	anti-adipogenic	in vitro	BMSCs from mice	(Wang et al. 2018)
lncRNA-H19/miR-130a/PPARγ	pro-lipid accumulation	in vitro and in vivo	mouse model of NFALD, HepG2 and Huh-7 cells for NAFLD cellular model	(Liu et al. 2019)
lncRNA-H19/PTBP1/SREBP1c	pro-lipid accumulation	in vitro and in vivo	human liver specimens, lncRNA-H19-deleted mice, lncRNA-H19 overexpressed and PTBP1 knockdown mice, primary hepatocytes from mice	(Liu et al. 2018)
lncRNA-H19/MBD1/PEGs	pro-adipogenic, anti-lipid accumulation	in vitro and in vivo	adipose tissues from mice exposed cold or exposed to chronic HFD feeding, PIBA cell line	(Schmidt et al. 2018)
lncRNA-H19/miR-130b	pro-lipid accumulation, pro-inflammatory	in vitro	blood samples from atherosclerotic patients and non-atherosclerotic patients, mouse macrophages cell line (Raw264.7)	(Han et al. 2018)
lncRNA-H19/p38&p65/NF-κB	pro-inflammatory	in vitro	blood samples from atherosclerotic patients and healthy volunteers, atherosclerotic plaque and adjacent tissues from ApoE^−/−^ mice, VSMCs, HUVECs	(Pan 2017)
		in vitro	blood samples from atherosclerotic patients and healthy volunteers, HUVECs	(Li et al. 2019a)
lncRNA-H19/let-7a/IL-6	pro-inflammatory	in vivo and ex vivo	human AAA samples and adjacent normal aortic tissues, mouse model of AAA, ApoE^−/−^ mice, mouse aortic VSMCs, mouse macrophages cell line (Raw264.7)	(Sun et al. 2019b)
lncRNA-H19/let-7/periostin	pro-inflammatory	in vitro	HUVECs	(Cao et al. 2019)
lncRNA-H19/ACP5	pro-proliferation, anti-apoptosis	in vitro and in vivo	blood samples from atherosclerotic patients and healthy subjects, mouse model of ischemic stroke, VSMCs, HUVECs	(Huang et al. 2019)
lncRNA-H19/let-7a/cyclin D1	pro-proliferation	in vitro and in vivo	common carotid arteries from rat model of carotid artery balloon injury, HA-VSMCs, HEK 293 T cells	(Sun et al. 2019c)
lncRNA-H19/let-7b/AT_1_R	pro-proliferation	in vitro and in vivo	Serum, lung tissues and PAMSCs from rat/mouse model of PAH	(Su et al. 2018)
lncRNA-H19/miR-148b/WNT/β-catenin	pro-proliferation, anti-apoptosis	in vitro	blood samples from atherosclerotic patient without any treatment and healthy volunteers, HA-VSMCs	(Zhang et al. 2018a)
lncRNA-H19/miR-675/PTEN	pro-proliferation	in vitro and in vivo	common carotid arteries from rat model of carotid artery balloon injury, HA-VSMCs, HEK 293 T cells	(Lv et al. 2018)
lncRNA-H19/miR-675-5p/Mfn2	pro-proliferation, anti-apoptosis	in vitro and in vivo	vascular walls from rat model of carotid artery balloon injury, VSMCs	(Xu and Sun 2018)
lncRNA-H19/HIF1α/Mdm2/p53/Bcl-2&BAX	pro-apoptosis	in vitro and in vivo	AAA samples from patients and abdominal aortic samples from organ donor controls, mouse model of AAA, mini-pigs model of AAA, HA-VSMCs	(Li et al. 2018)
lncRNA-H19/miR-22-3p/KDM3A	alleviate MI	in vitro and in vivo	rat model of AMI, neonatal rat cardiomyocytes	(Zhang et al. 2020)
lncRNA-H19/miR-139/Sox8	alleviate MI	in vitro	rat embryonic cardiomyocyte cell line (H9C2)	(Gong et al. 2017)
lncRNA-H19/YB-1	aggravate cardiac remodeling after infarction	in vitro and in vivo	mouse model of MI, cardiomyocytes and cardiac fibroblasts from MI mice, mouse embryonic fibroblast cell line (NIH3T3)	(Choong et al. 2019)
lncRNA-H19/miR-877-3p/Bcl-2	alleviate myocardial I/R	in vitro and in vivo	mouse model of myocardial I/R injury, NMVCs	(Li et al. 2019b)
lncRNA-H19/miR-103&miR-107/FADD	alleviate myocardial I/R	in vitro and in vivo	mouse model of myocardial I/R injury, rat embryonic cardiomyocyte cell line (H9C2)	(Wang et al. 2015)
lncRNA-H19/nucleolin	alleviate myocardial I/R	in vitro and in vivo	mouse model of myocardial IP and I/R injury, rat embryonic cardiomyocyte cell line (H9C2), neonatal rat cardiomyocytes	(Chen et al. 2020)
lncRNA-H19/miR-29b-3p/cIAP1	alleviate myocardial I/R	in vitro	neonatal rat cardiomyocytes, rat embryonic cardiomyocyte cell line (H9C2), HEK 293 T cells	(Zhang et al. 2019)
lncRNA-H19/miR-675/PPARα	aggravate myocardial I/R	in vitro and in vivo	mouse model of myocardial I/R injury, neonatal mouse cardiomyocytes	(Luo et al. 2019)

*Abbreviations*: *lncRNAs* long non-coding RNAs, *miRNAs* microRNAs, *CTCF* CCCTC-binding factor, *PKD1* Polycystic kidney disease 1, *ApoE* Apolipoprotein E, *VEGF* Vascular endothelial growth factor, *BMSCs* Bone marrow mesenchymal stem cells, *CNV* Corneal neovascularization, *HUVECs* Human umbilical vein endothelial cells, *eNOS* endothelial NO synthase, *HMEC-1* Human dermal vascular endothelial cells, *EZH2* Enhancer of zeste homolog 2, *VASH1* Vasohibin1, *HAMSCs* Human amniotic mesenchymal stem cells, *STAT3* Signal transducer and activator of transcription 3, *hCoAEC* human coronary artery endothelial cells, *Class II HDAC* Class II histone deacetylase, *LCoR* Ligand-dependent corepressor, *PPARγ* Peroxisome proliferator-activated receptor γ, *NFALD* Nonalcoholic fatty liver disease, *PTBP1* Polypyrimidine tract-binding protein 1, *SREBP1c* Sterol regulatory element-binding protein 1c, *MBD1* Methyl-CpG–binding domain protein 1, *HFD* High-fat diet, *PIBA* Primary immortalized brown adipocytes, *NF-κB* Nuclear factor-κB, *VSMCs* Vascular smooth muscle cells, *IL-6* Interleukin-6, *AAA* Abdominal aortic aneurysm, *ACP5* Acid phosphatase 5, *HA-VSMCs* Human aortic vascular smooth muscle cells, *HEK* Human embryonic kidney, *AT*_*1*_*R* Ang II type 1 receptor, *PASMCs* Pulmonary arterial smooth muscle cells, *PAH* Pulmonary arterial hypertension, *PTEN* Phosphatase and tensin homology deleted on chromosome ten, *Mfn2* Mitofusin-2, *HIF1α* Hypoxia-inducible factor 1α, *Mdm2* Mouse double minute 2 homolog, *Bcl-2* B cell lymphoma 2, *BAX* Bcl-2 associated X, *KDM3A* Lysine (K)-specific demethylase 3A, *MI* Myocardial infarction, *AMI* Acute myocardial infarction, *Sox8* Sex determining region Y (SRY)-related high-mobility group box 8, *YB-1* Y-box-binding protein-1, *I/R* Ischemia/reperfusion, *NMVCs* Neonatal mouse ventricular cells, *FADD* Fas-associated protein with death domain, *IP* Ischemic preconditioning, *cIAP1* cellular inhibitor of apoptosis protein 1, *PPARα* Peroxisome proliferator-activated receptor α

**Fig. 2:**
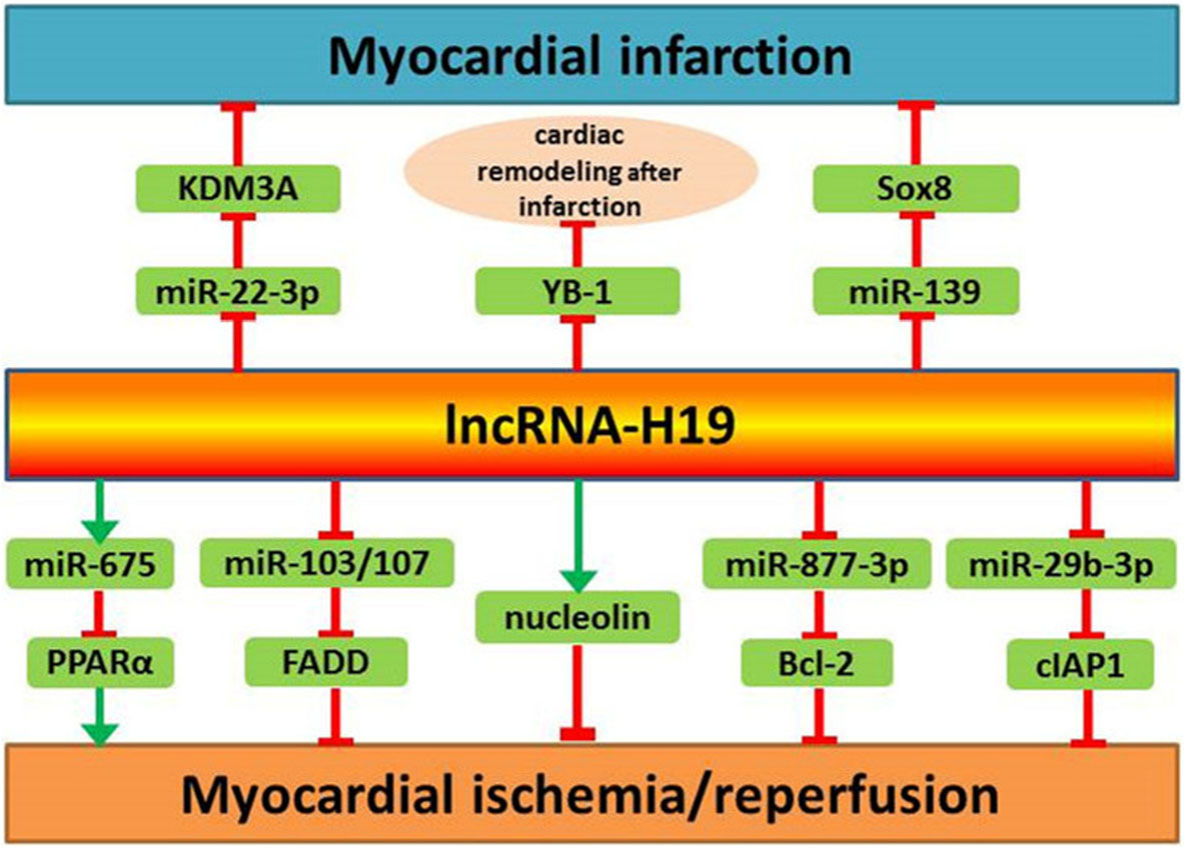
The role of lncRNA-H19 and its targets in cardiomyocyte injury induced by ischemia/reperfusion or infarction.: aggravate,: alleviate

Compared with the normal healthy control subjects, increased levels of lncRNA-H19 have been detected in the serum of atherosclerosis patients. Hence, the serum levels of lncRNA-H19 could be proposed as risk factors or diagnostic markers for atherosclerosis (Pan 2017; Huang et al. 2019; Han et al. 2018; Li et al. 2019a; Zhang et al. 2018a). Studies have shown that lncRNA-H19 upregulates VEGF-A by targeting miR-199a-5p, miR-29a and miR-29c. Thus, it can be inferred that lncRNA-H19 promotes angiogenesis by regulating multiple targets (Hou et al. 2018; Jia et al. 2016; Sun et al. 2019a). However, the exact role of lncRNA-H19 in adipogenesis and lipid metabolism remains to be elucidated. Ongoing studies should further clarify its relationship with different kinds of tissue and cells such as adipose tissue and precursor cells of adipocytes. With regard to the atherosclerosis-related inflammatory response, current studies have shown pro-inflammatory effects of lncRNA-H19 through several mechanisms including the NF-κB signaling pathway. Although the exact mechanisms are complicated, there is compelling evidence that lncRNA-H19 plays a critical role in the proliferation and apoptosis of VSMCs and ECs. Moreover, it has been shown that lncRNA-H19 is involved in cardiomyocyte injury induced by I/R injury or infarction (Fig. 2). Additionally, knockdown of lncRNA-H19 inhibits abnormal differentiation of small intestinal epithelial cells in diabetic mice and elevated hepatic expression of lncRNA-H19 contributes to hyperglycemia in type 2 diabetes, suggesting its potential role in the modulation of type 2 diabetes (Zhang et al. 2018b; Shan et al. 2018).

It is important to note that lncRNA-H19 mainly exerts its function by acting as a ceRNA to sponge miRNA, generating miRNA or directly regulating the downstream target. Experimental results have suggested that one miRNA could target more than 100 mRNAs and one miRNA could be targeted by many upstream factors (Chen et al. 2013; Najafi-Shoushtari et al. 2010; Rayner et al. 2010). Thus, how the single lncRNA-H19 regulates multiple targets in vitro and in vivo should be precisely identified in ongoing studies.

In summary, many studies currently suggest the pathophysiological contribution of lncRNA-H19 in the process of atherosclerosis. Based on increasing evidence, there has been a good reason to propose that lncRNA-H19 may serve as a potential indicator or a novel target for developing therapeutic strategies for atherosclerosis. In the future, much more research will be necessary before novel lncRNA-H19-based therapeutics are utilized into clinical practice.

## Future perspectives and challenges

Many questions have been raised from the current studies about lncRNA-H19. It will be necessary to perform further studies to elucidate the following questions: (i) What influences lncRNA-H19 levels in the onset, development and progression of atherosclerosis? (ii) How does lncRNA-H19 strictly regulate multiple targets in vitro and in vivo? (iii) Are there any other lncRNA-H19 targets that can increase the risk of atherosclerosis? (iv) Does lncRNA-H19 influence the process of atherosclerosis by targeting different pathways in various cell types and tissues? (v) Why and when lncRNA-H19 may serve pro-apoptotic vs. anti-apoptotic roles and pro-survival vs. deleterious roles in I/R injury? Note that there are still some technical challenges that make future lncRNA-H19-based therapeutics difficult. Importantly, the potential significance of lncRNA-H19 requires careful studies that complement the development of reliable strategies to specifically target different genes and proteins in atherosclerosis-related cells. Understanding the molecular mechanisms and cellular pathways controlled by lncRNA-H19 will be warranted.

## Data Availability

Not applicable.

## Abbreviations

AS: Atherosclerosis
MI: Myocardial infarction
lncRNAs: Long non-coding RNAs
miRNAs: microRNAs
PRC2: Polycomb repressive complex 2
ceRNAs: Competing endogenous RNAs
VSMCs: Vascular smooth muscle cells
IGF2: Insulin-like growth factor 2
ECs: Endothelial cells
VEGF: Vascular endothelial growth factor
PlGF: Placenta growth factor
MMP-2: Matrix metalloproteinase 2
CTCF: CCCTC-binding factor
PKD1: Polycystic kidney disease 1
miR-199a-5p: microRNA-199a-5p
HMEC-1: Human dermal vascular endothelial cells
eNOS: Endothelial NO synthase
JNK: C-Jun N-terminal kinase
AMPK: AMP-activated protein kinase
HAMSCs: Human amoiotic mesenchymal stem cells
EZH2: Enhancer of zeste homolog 2
VASH1: Vasohibin1
STAT3: Signal transducer and activator of transcription 3
WAT: White adipose tissue
BAT: Brown adipose tissue
PVAT: Perivascular adipose tissue
BMSCs: Bone marrow mesenchymal stem cells
HDAC: Histone deacetylase
LCoR: Ligand-dependent corepressor
TG: Triglycerides
TC: Total cholesterol
LDL-C: Lipoprotein-cholesterol
HDL-C: High density lipoprotein-cholesterol
NAFLD: Nonalcoholic fatty liver disease
PPARγ: Peroxisome proliferator-activated receptor γ
SREBP1c: Sterol regulatory element-binding protein 1c
PTBP1: Polypyrimidine tract-binding protein 1
MBD1: Methyl-CpG–binding domain protein 1
PEGs: Paternally expressed genes
LPS: Lipopolysaccharide
NF-κB: Nuclear factor-κB
MAPK: Mitogen-activated protein kinase
HUVECs: Human umbilical vein endothelial cells
IL-6: Interleukin-6
AAA: Abdominal aortic aneurysm
ox-LDL: Oxygenized low density lipoprotein
ACP5: Acid phosphatase 5
AT_1_R: Ang II type 1 receptor
HA-VASC: Human aortic vascular smooth muscle cell
PTEN: Phosphatase and tensin homology deleted on chromosome ten
Mfn2: Mitofusin-2
HIF1α: Hypoxia-inducible factor 1α
Mdm2: Mouse double minute 2 homolog
Bcl-2: B cell lymphoma 2
BAX: Bcl-2 associated X
Hcy: Homocysteine
I/R: Ischemia/reperfusion
AMI: Acute myocardial infarction
CK: Creatine kinase
KDM3A: Lysine (K)-specific demethylase 3A
Sox8: Sex determining region Y (SRY)-related high-mobility group box 8
YB-1: Y-box-binding protein-1
ECM: Extracellular matrix
FADD: Fas-associated protein with death domain
H/Post: Hypoxic postconditioning
cIAP1: Cellular inhibitor of apoptosis protein 1
